# Loss of peroxiredoxin 6 alters lipid composition and distribution resulting in increased sensitivity to ferroptosis

**DOI:** 10.1042/BCJ20240445

**Published:** 2024-12-23

**Authors:** Daniel J. Lagal, Ángel Ortiz-Alcántara, José R. Pedrajas, Brian McDonagh, J. Antonio Bárcena, Raquel Requejo-Aguilar, C. Alicia Padilla

**Affiliations:** 1 Department of Biochemistry and Molecular Biology, University of Córdoba, Cordoba, Spain; 2 Maimónides Biomedical Research Institute of Córdoba (IMIBIC), Cordoba, Spain; 3 Group of Biochemistry and Cell Signaling in Nitric Oxide, Department of Experimental Biology, University Institute of Research in Olive Groves and Olive Oils, University of Jaén, Jaen, Spain; 4 Discipline of Physiology, School of Medicine, University of Galway, Galway, Ireland

**Keywords:** ferroptosis, lipid droplet, lipid peroxidation, lipid raft, lysophospholipids, phospholipase A2

## Abstract

Peroxiredoxin 6 (PRDX6) is a multifunctional enzyme involved in phospholipid peroxide repair and metabolism. In this study we investigated the global lipid composition of a human hepatocarcinoma cell line SNU475 lacking PRDX6 and lipid related cellular processes. There was a general decrease in multiple lipids species upon loss of PRDX6, in particular sphingomyelins and acylcarnitines, consistent with previously observed alterations in cell signaling pathways and mitochondrial dysfunction. Deprivation of docosahexaenoic acid and related species was also evident. However, a few striking exceptions are worth highlighting: (1) Three specific arachidonic acid (AA) containing phophatidylcholines (PC) increased significantly. The increase of sn1-stearic/sn2-PUFA containing PC and sn2-AA containing plasmenyls are indicative of a preference of PRDX6 iPLA2 activity for these AA storage glycerophospholipids. (2) Several polyunsaturated fatty acids (PUFA) and PUFA containing triacylglycerols accumulated together with increased formation of lipid droplets, an indication of altered FA flux and PUFA sequestration in PRDX6 knockout cells. Loss of PRDX6 resulted in increased sensitivity to erastin-induced ferroptosis, independent of selenium and GPX4, as a consequence of increased levels of lipid hydroperoxides, that reverted to normal levels upon rescue with PRDX6. The results presented demonstrate that all three enzymatic activities of PRDX6 contribute to the role of this multifunctional enzyme in diverse cellular processes, including membrane phospholipid remodeling and glycerophospholipid functional diversity, resulting in altered lipid peroxides and modulation of AA disposition and traffic. These contributions highlight the complexity of the changes that loss of PRDX6 exerts on cell functionality.

## Introduction

The composition of cells is highly dominated by lipids [1], which are the fundamental building blocks of biological membranes. Aside from this structural role, lipids also play multiple important functions in biological systems as energy storage entities and participating in metabolic regulation, membrane trafficking, signaling, proliferation or cell death as apoptosis or ferroptosis.

Membrane organization dependent on lipid composition is critical for cell function and its targeting is an attractive therapeutic strategy against a wide range of diseases, including cancer [2,3]. Membrane organization affects transduction, plasticity and trafficking and regulates intracellular signaling, redox balance and cell death. Small, heterogeneous and highly dynamic lipid domains in the cell membrane rich in sphingolipids and cholesterol, so called lipid rafts [4], have been related to signaling pathways involved in cancer progression; they can recruit or exclude signaling proteins, including kinases and phosphatases in response to external and intracellular stimuli leading to dynamic changes in cell size and composition [5,6].

Peroxiredoxin 6 (PRDX6) is unique among peroxiredoxins (Prdx) as it acts as a peroxidase on phospholipid hydroperoxides [7] and possesses two additional functions, Ca^2+^ independent phospholipase A2 (iPLA2) [8] and lysophosphatidylcholine acyl transferase (LPCAT) activities [9]. Several studies have shown that PRDX6 plays an important role in membrane repair thanks to its panoply of activities. The peroxidase activity it can reduce phospholipid hydroperoxides to alcohols, while its other two activities it can eliminate phospholipid hydroperoxides at sn-2 position and replace them by their reduced form. PRDX6 has been linked to various pathologies including cancer [10,11]. Elevated levels of PRDX6 have been described in a wide variety of human cancers and its overexpression promotes invasion and metastasis in lung cancer [12] and lung tumor growth in mice through activation of JAK2/STAT3 signaling [13]. PRDX6 has also been implicated in the pathogenesis of inflammatory, metabolic and neurodegenerative diseases, as well as in ocular damage and male infertility [11]. A link between PRDX6 and insulin signaling pathways has also been demonstrated, that can influence glucose homeostasis, lipid metabolism and inflammation has been documented [10]. Underlying this multifunctional role of PRDX6 is its peculiar activities involving lipids homeostasis and the importance role of lipids in cell signaling [14,15].

In a previous study, we observed that loss of PRDX6 in the hepatocarcinoma cell line SNU475 provokes metabolic reprogramming, mitochondrial dysfunction, cell cycle arrest, cytoskeleton alteration, MMP2 inhibition, which drastically affect cell proliferation, migration and invasive potential [16]. Our proteomic study in cells lacking PRDX6 indicated inhibition of the regulatory role of lysophosphatidylcholine (LPC) with down-regulation of fatty acid synthase and cholesterol biosynthesis. An increase in peroxidized lipids was also observed and we posit that elevated levels of fatty acid hydroperoxides (HpFA) would increase oxidative damage and initiate cell dysfunction. The accumulation of hydroperoxide phospholipids derived from the peroxidized cell membrane can activate ferroptosis [17]. Similarly, it has been demonstrated that inhibition of iPLA2β activity in tumor cells induces ferroptosis in a GPX4-independent manner and promotes p53-dependent tumor suppression in xenograft mouse models [18].

The involvement of PRDX6 in rat hepatic lipid metabolism has also been described [19]. Furthermore, lower levels of oxylipins, specifically 13-hydroxylinoleic acid ((H)LA), in white adipose tissue of PRDX6 null mice have been reported [20]. It was suggested that loss of PRDX6 peroxidase activity eventually results in a decrease in fatty acid esters of hydroxy fatty acids, while loss of iPLA2 activity resulted in only minor effects [20].

An analytical approach was used to study possible effects due to loss of PRDX6 iPLA2 activity: in the absence of PRDX6 iPLA2 activity, the substrates of the enzyme would increase while the products would decrease. In the case of PRDX6 deficiency, its peroxidase and iPLA2 substrates, fatty acid hydroperoxides (HpFA) and phospholipids (PL) would accumulate, whereas its products hydroxy-fatty acids (HFA) and lysophospholipids (LPL) would decrease. Changes in any of these metabolites should be considered for the identification of individual triggers of the cell response to loss of PRDX6.

In this study we performed a comparative analysis of the global lipidome of SNU475 standard cells and cells lacking PRDX6. The lipid composition upon loss of PRDX6 changed markedly affecting lipid droplet (LD) formation, sensitivity to ferroptosis and stability of lipid rafts. These remarkable alterations underlie the drastic phenotypic changes following loss of PRDX6 observed by our group that affect cell proliferation, migration and invasion.

## Results

### Determination of the differential lipidome

**Figure 1.**
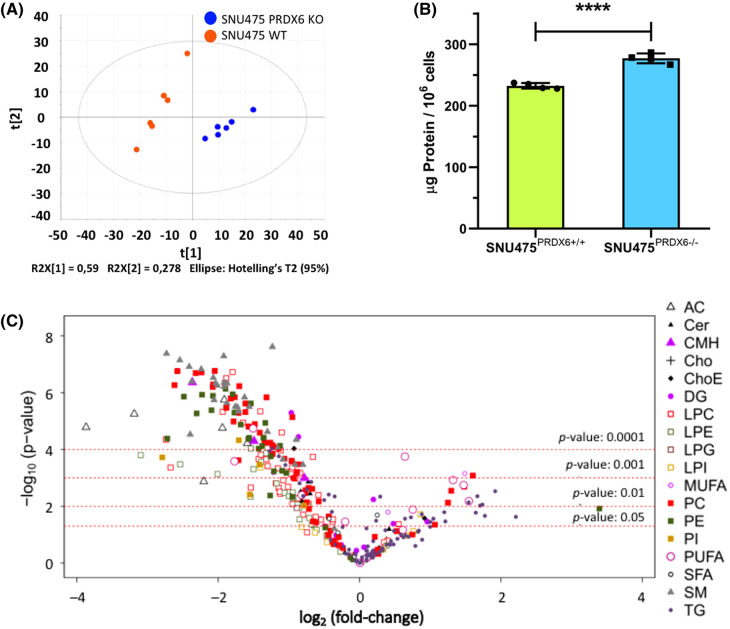
Differential lipidomic analysis between SNU475-WT and SNU475-KO cells. (**a**) Score scatter plot of the PCA model of SNU475 samples. The ellipse represents 95% confidence interval according to Hotelling's T2 test. This score scatter plot reveals a clear separation between the experimental groups as effect of the PRDX6 deletion. (**b**) Comparison of protein content between SNU475^PRDX6+/+^ and SNU475^PRDX6−/−^ cells. Protein concentration was determined in cells of different passages as described in Materials and Methods. Unpaired Student's *t*-test, ****P-value ≤ 0.0001. Individual values and SD bars are also shown (*n* = 4). (**c**) Volcano plot [−log_10_(*P*-value) vs. log_2_(fold-change)] for the comparison between PRDX6-KO and PRDX6-WT hepatocellular carcinoma SNU475 cells. AC, acyl-carnitines; Cer, ceramides; CMH, monohexosylceramides; Cho, cholesterol; ChoE, cholesteryl esters; DG, diglycerides; LPC, LysoPC; LPE, lysoPE; LPI, LysoPI; MUFA, monounsaturated-FA; PC, phosphatidylcholines; PE, phosphatidylethanolamines; PI, phosphatidylinositols; PUFA, polyunsaturated-FA; SFA, saturated-FA; SM, sphingomyelins; TG, triglycerides.

A wide range of different lipid subclasses comprising 351 accurately detected lipids were quantified in the analyzed SNU475 cells. Differences between SNU475^PRDX6+/+^ and SNU475^PRDX6−/−^ genotypes were assessed by multivariate data analysis revealing a clear separation between the two experimental groups as an effect of PRDX6 deletion as shown in the scatter plot in Figure 1A. The absence of PRDX6 has a notable effect on the lipidome of the hepatocarcinoma SNU475 cells, demonstrating that PRDX6-KO cells had lower levels of almost all the lipid species than PRDX6-WT cells, especially glycerophospholipids, acyl-carnitines (ACs) and sphingomyelins, despite the fact that the cells are larger and their protein content was greater [16] (Figure 1B). Moreover, the opposite trend was found for several triglycerides, diglycerides, and fatty acids (Figure 1C).

These differences were confirmed in the univariate data analysis. The changes observed in fatty acids (FA) due to the lack of PRDX6 did not show a clear trend, a few monounsaturated-FA (MUFA) and PUFA increased but one MUFA and several PUFA decreased (Table 1). Lipid classes markedly affected by the lack of PRDX6 are analyzed in the following sections.

**Table 1.** Changes in the levels of fatty acids.

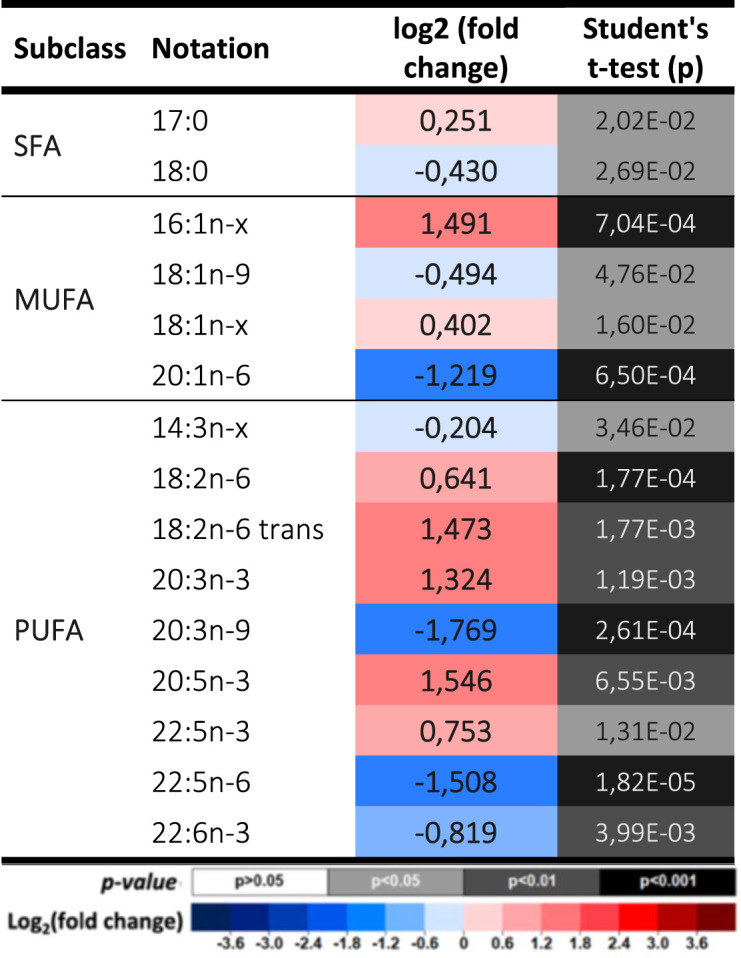

Heatmap representing statistically significant (*P* ≤ 0.05) binary comparisons between PRDX6-KO and PRDX6-WT SNU475 cells per free fatty acids. Color codes are indicated at the bottom of the figure. Blue/red sections denote lipids that were reduced or increased in PRDX6-KO cells, respectively; gray/black bars indicate significant changes (light-gray, *t*-test *P*-value < 0.05; dark-gray, *P* < 0.01; black, *P* < 0.001). Abbreviations as in Figure 1.

### The absence of PRDX6 induces selective decrease of docosahexaenoic acid and redistribution of arachidonic acid

Our results demonstrate that all docosahexaenoic acid (DHA) containing glycerophospholipids decrease drastically in the absence of PRDX6 and even free DHA diminished (Table 2).

**Table 2.** Docosahexaenoic acid (DHA) containing lipids.

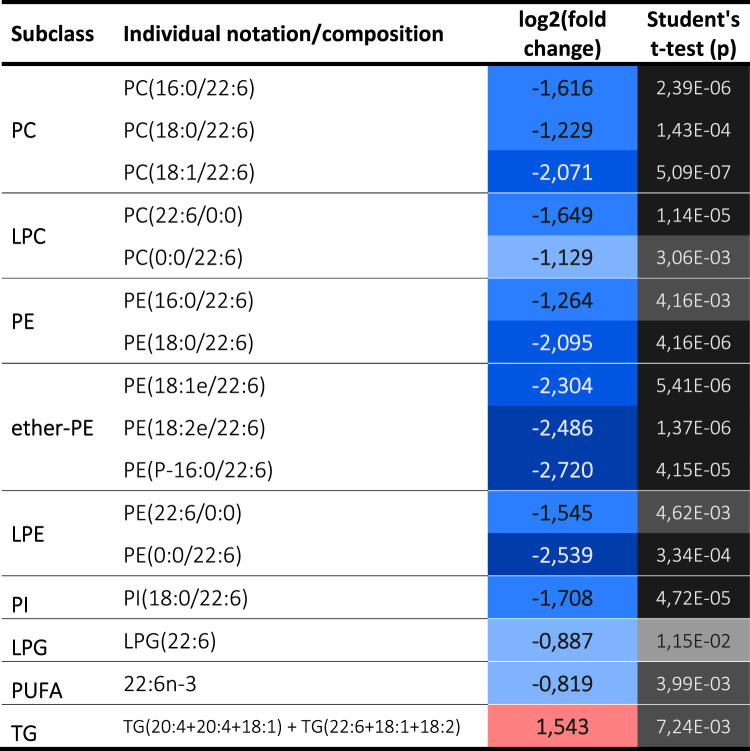

Heatmap representing binary comparisons between PRDX6-KO and PRDX6-WT SNU475 cells per DHA (22:6*n*−3) containing lipids. Color codes as in Table 1 and abbreviations as in Figure 1.

The levels of arachidonic acid (AA) were also drastically decreased in all the glycerophospholipid classes, but with the striking exception of three phosphatidylcholine (PC) containing species (Table 3) which may be indicative of specificity of the phospholipase A2 activity of PRDX6 for these lipids with AA at sn2 position. However, the presence of AA increased markedly in triglycerides, a likely indication of alteration in its metabolism and trafficking.

**Table 3.** Arachidonic acid (AA) containing lipids.

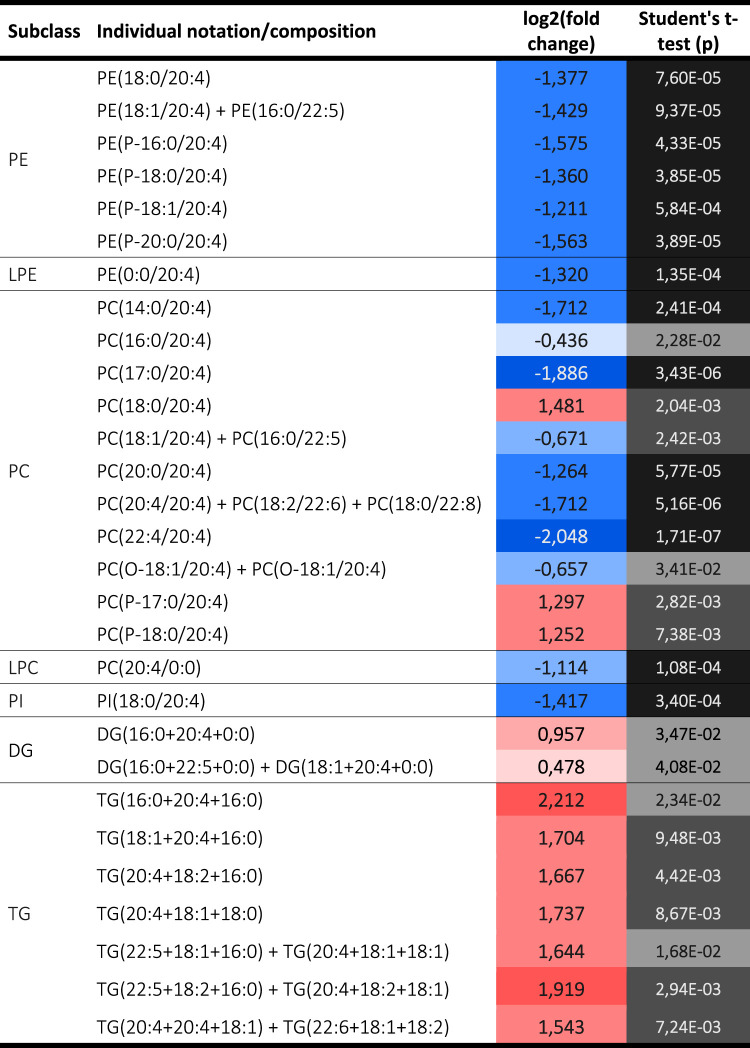

Heatmap representing binary comparisons between PRDX6-KO and PRDX6-WT SNU475 cells per AA (20:4) containing lipids. Color codes as in Table 1 and abbreviations as in Figure 1.

These changes point to the existence of a specific relationship between these two biologically active PUFAs, and PRDX6 enzymatic activities.

### Changes in sphingomyelins, ACs, triglycerides and LDs

Sphingomyelins decrease dramatically in hepatocarcinoma SNU475 cells lacking PRDX6 (Figure 1) that together with the increase in PUFA containing PC phospholipids (see section ‘Sensitivity to ferroptosis’ below), would suggest an alteration in the stability of lipid rafts, which is critical in cancer development and progression.

**Figure 2.**
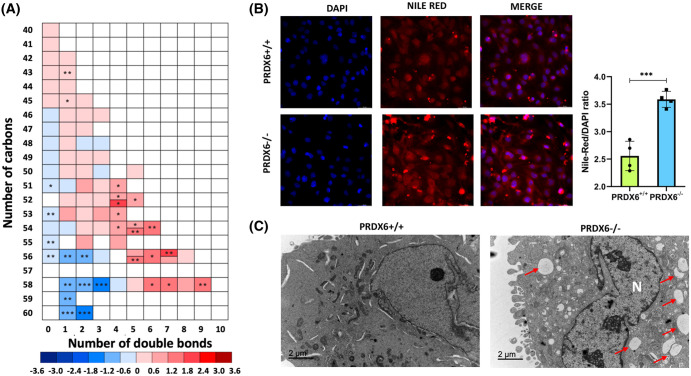
Changes of triglycerides and presence of droplets. (**a**) Heatmap representation of the influence of the number of carbons and double bonds in changes of triglycerides for the comparison PRDX6 KO vs. PRDX6 WT. The ‘*y*’ axis denotes the total number of carbons and the ‘*x*’ axis the number of double bonds per TG. Color scale indicates the log_2_ (fold-changes): boxes in blue denote reduced levels in PRDX6 KO cells, while boxes in red denote increased levels. Split boxes indicate two compositional isomers with different statistical significance. Student's *t*-test *P*-values: *P < 0.05, **P < 0.01; ***P < 0.001. **(b**) Nile red staining of PRDX6-KO and PRDX6-WT SNU475 cells. Representative images of SNU475^PRDX6+/+^ and SNU475^PRDX6−/−^ cells are shown. Intracellular lipid droplets in cells are stained red and the nuclei are stained blue; levels of fluorescence for Nile red were measured in the two cell types and plotted in the graph shown with SD and statistical significance (*n* = 4, corresponding ‘*n*’ to 4 different culture dishes); (**c**) transmission electron microscopy of SNU475^PRDX6+/+^ and SNU475^PRDX6−/−^ cells; representative micrographs are shown; note the marked increase of LDs (red arrows) in PRDX6-KO cells compared with WT cells. N, nucleus.

All the AC species detected decreased sharply in SNU475^PRDX6−/−^ cells (Figure 1). This decrease could be related to the lack of acyl-CoA entering the mitochondria, which is not surprising given the diminished respiratory capacity of SNU475^PRDX6−/−^ cells [16]. This could also be the reason why triglycerides (TG) accumulate in cells lacking PRDX6 (Figure 1). Most TG with short acyl chains (50 carbons or less) remained unchanged in the comparison. However, interestingly, a different pattern was observed in TG with longer acyl chains: highly polyunsaturated species (containing at least four double bonds) increased in SNU475 cells lacking PRDX6, coherent with an increased level of long chain fatty acid CoA ligase ACSL3 [16], while saturated or unsaturated species with three or fewer double bonds decreased (Figure 2A). A greater number of LDs were observed in PRDX6-KO cells (Figure 2B,C) where a large amount of PUFAs can be sequestered.

### Glycerophospholipids profile indicates substrate specificity of PRDX6 iPLA2 activity

**Figure 3.**
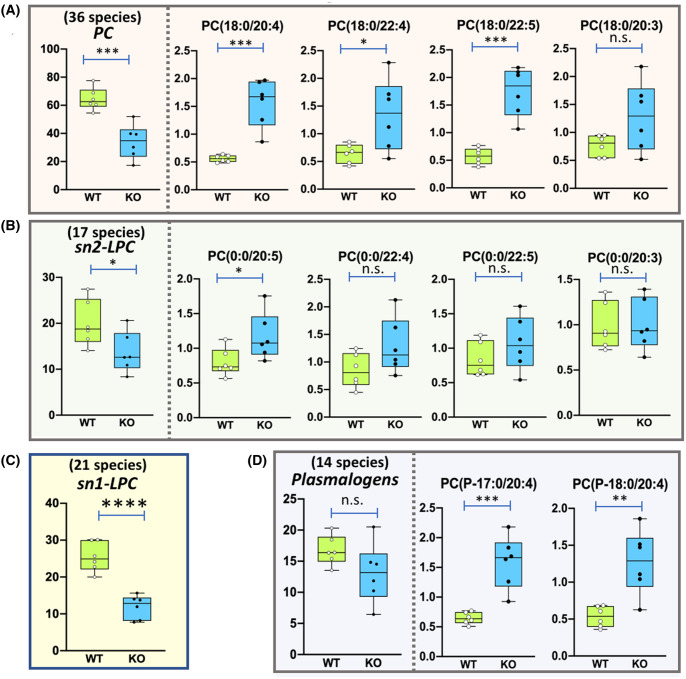
Phosphatidylcholine glycerophospholipids levels in SNU475^PRDX6+/+^ versus SNU475^PRDX6−/−^ cells. (**a**) Diacylglycerophosphocholine (PC); (**b**) 2-Monoacylglycerophosphocholine (sn2-LPC); (**c**) 1-Monoacylglycerophosphocholine (sn1-LPC); (**d**) Plasmalogens. The plot on the left hand side of the vertical dotted line in panels A, B and D shows the average level of the corresponding species, including the number of compounds detected and quantified; the plots on the right hand side of the dotted line shows the levels of those compounds that behave opposite the average.

Phospholipase A2 activity catalyzes the hydrolysis of the sn-2 fatty acyl ester bond of glycerophospholipids to produce free fatty acids and sn-1 LPL [21]. PRDX6 has been reported to have iPLA2 activity [22], and in particular a preference for phosphatidylcholines as substrates, with some specificity for AA at the sn-2 position [12,23–27]. The complete profile of glycerophospholipids (192 species) was diminished with the loss of PRDX6 (Figure 1). Among glycerophosphatidylcholines (PC, 67 species), PC with stearic acid at sn1 and long chain PUFA at sn2: PC(18:0/20:4), PC(18:0/22:4) and PC(18:0/22:5) were remarkable exceptions (Figure 3A). In the case of LPL, 12 out of 17 species of sn2-LPC decreased, but 4 species with long chain PUFA did not (Figure 3B). Moreover, the complete set of sn1-LPC (21 species) was diminished (Figure 3C).

All these specific changes are coherent with the loss of PLA2 activity of PRDX6 with substrate specificity for PC containing stearic acid at sn1 position and long chain PUFA at sn2. Contrary to PC phospholipids, the equivalent PE and LPE species did not behave in the same way (Supplementary Table S1), a likely indication that PE phospholipids are not substrates for PLA2 activity of PRDX6. Few changes were detected in LPI, the only major change was the diminution of LPI (18:1) in SNU475^PRDX6−/−^ cells (Supplementary Table S1). The effect of loss of PRDX6 on ether-linked glycerophospholipids (plasmalogens) levels deserves special attention.

### Loss of PRDX6 in SNU475 cells disrupts plasmalogen homeostasis

Most of the ether-linked glycerophospholipids (51 species), either as ether-linked (plasmanyl) or vinyl-ether linked (plasmenyl), were also reduced in SNU475^PRDX6−/−^ cells. Remarkably, among the plasmenyls, only two species increased: PC(P-17:0/20:4) and PC(P-18:0/20:4), both are canonical plasmalogens with AA esterified at the sn2 position (Figure 3D). The data demonstrate that loss of PRDX6 in SNU475 cells disrupts plasmalogen homeostasis that could be in the root of the signaling, metabolic and cell cycle changes we have previously observed in cells devoid of PRDX6 [16,25,28].

Plasmalogen remodeling is limited to the *sn-2* position by rapid deacylation-reacylation steps, as occurs in the ester analogues, catalyzed by the very large PLA2 superfamily that also drives the release of AA. Coincidentally, the levels of AA were drastically decreased in the absence of PRDX6 in all the glycerophospholipid classes, except for three phosphatidylcholine containing species, two of which were the plasmalogens described above (Table 3). Contrary to glycerophospholipids, the levels of AA increased markedly in triglycerides, a likely indication of alteration of its metabolism.

The correlation between the lack of PRDX6 and the accumulation of AA containing plasmalogens in SNU475^PRDX6−/−^ cells suggests that these lipid molecules could also be substrates of PRDX6 PLA2 activity.

### Lack of PRDX6 makes SNU475 cells more sensitive to ferroptosis

**Figure 4.**
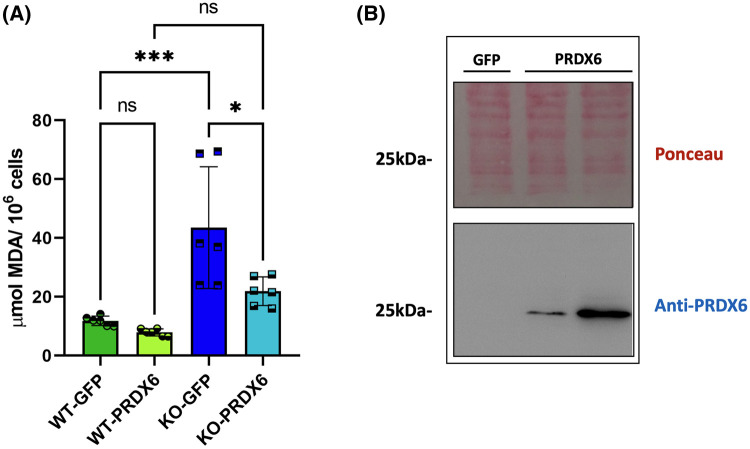
Expression of PRDX6 reduces lipid peroxidation in SNU475 cells lacking PRDX6. (**a**) Lipid peroxidation was determined by quantifying MDA levels per number of cells using the TBARS assay in SNU475^PRDX6+/+^ (WT) and SNU475^PRDX6−/−^ (KO) cells transfected with 2.4 µg plasmid expressing either PRDX6 (WT-PRDX6; KO-PRDX6) or GFP (WT-GFP, KO-GFP). One-way ANOVA, *P-value ≤ 0.05; ***P-value ≤ 0.001. (**b**) Transfection efficiency in SNU475^PRDX6−/−^ cells was checked measuring the abundance of PRDX6 72 h post-transfection: Ponceau-Red staining and Western blot against PRDX6; lanes 1, 2 and 3 correspond to SNU475^PRDX6−/−^ cells transfected with 2.4 µg GFP-containing plasmid (lane 1) and 1.2 and 2.4 µg of PRDX6-containg plasmid (lanes 2 and 3).

**Figure 5.**
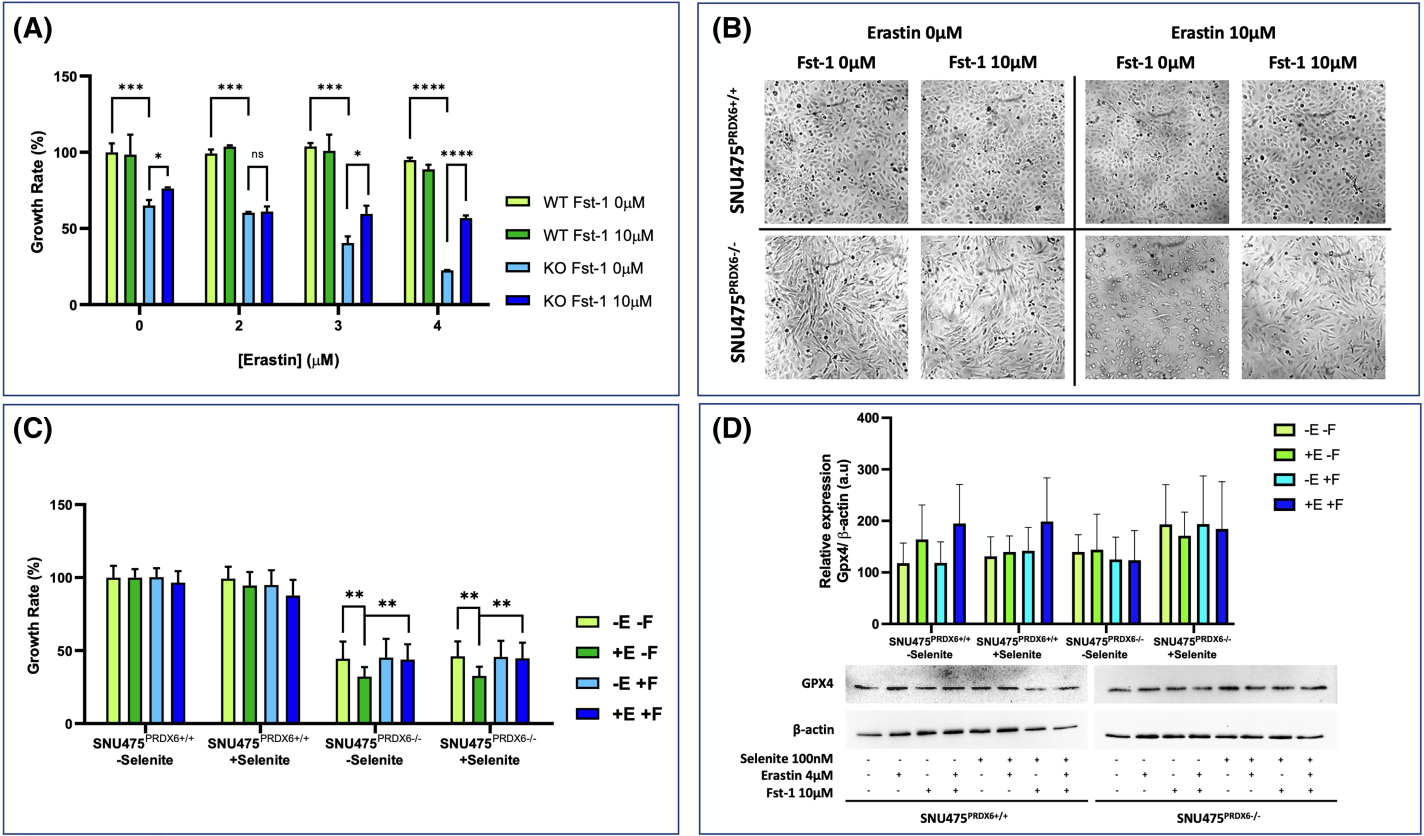
Erastin induced ferroptosis and prevention by ferrostatin in SNU475 cells lacking PRDX6. (**a**) The effect of different concentrations of erastin on cell viability was determined by SRB assay, as described in Materials and Methods section; relative cell viability is plotted for each cell type in percentage relative to their corresponding controls without erastin; WT (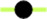, 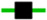) is shown in green and KO (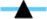, 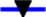) in blue; light colors represent erastin treatment and the preventive effect of simultaneous addition of 10 µM Fst-1 is shown in deep colors; SD bars are also shown (*n* = 4). (**b**) Representative images of SNU475^PRDX6+/+^ and SNU475^PRDX6−/−^ cells treated or not with erastin and ferrostatin-1 as indicated. (**c**) Effect of cell growth for 48 h with sodium selenite on the induction of ferroptosis with 4 µM erastin (deep green) and its prevention with 10 µM ferrostatin (deep blue) in WT and KO cells. (**d**) GPX4 expression was determined by Western blot (*n* = 4) with specific antibodies (1:1000) in cell extracts subjected to the same treatments as in **c**. Two-way ANOVA, *P < 0.05, **P < 0.01; ***P < 0.001; ****P < 0.0001; ns, not significant.

Ferroptosis is a type of cell death, that relies on iron accumulation and subsequent lipid peroxidation. We have previously described that the lack of PRDX6 increased the amount of peroxidized lipids [16,28]. Here we demonstrate that re-expression of PRDX6 in SNU475^PRDX6−/−^ cells reverted lipid hydroperoxides levels to the levels of wild type cells, indicating a role for PRDX6 in the protection or elimination of this lipid peroxidation (Figure 4A,B). Further studies are required to confirm if this is through its peroxidase activity that it can reduce lipid hydroperoxides and/or its iPLA2 and LPCAT activities that remove lipid hydroperoxides at the sn-2 position and replace them with their reduced form. Nevertheless, we have shown that increased peroxidized lipids due to the lack of PRDX6 was related to an increased sensitivity of the cells to erastin-induced ferroptosis. Erastin is an inhibitor of the cystine/glutamate antiporter (xCT) and of glutathione synthesis that can induce ferroptosis in cancer cells [29]. Our results demonstrate that cells containing PRDX6 were resistant to erastin-induced ferroptosis, while only 30% cell viability was observed in cells lacking PRDX6 relative to their control after 24 h of erastin treatment (Figure 5A). In both cases, a concentration of 10 µM ferrostatin-1 (Fst-1), a scavenger of initiating alkoxyl radicals [30], prevented erastin-induced ferroptosis (Figure 5A). Similar results of cell viability and cell death were observed after 48 and 72 h of erastin treatment (Supplementary Figure S1). Addition of erastin not only drastically reduced cell viability in SNU475^PRDX6−/−^ cells, but also induced conspicuous alterations of cell morphology, an indication of profound physiological changes, that were also prevented by Fst-1 (Figure 5B). These changes were observed directly in the culture plates both by light microscopy (Figure 5B), and by confocal microscopy with phalloidin labeling (Supplementary Figure S2). No relevant changes in mitochondrial structure were observed (Supplementary Figure S2), probably because cells lacking PRDX6 already have structurally and functionally highly affected mitochondria, as we have shown in previous work [16]. The selenoprotein GPX4 has previously been described to inhibit the induction of ferroptosis [31], however our results indicate that the induction of ferroptosis by erastin in SNU475^PRDX6−/−^ cells was independent of selenium (Figure 5C) and GPX4 (Figure 5D).

Our results demonstrate that loss of PRDX6 results in extensive lipid remodeling, cell morphology changes and sensitization to ferroptosis induced cell death. The response of these cells is to protect themselves from cell death by sequestering PUFA-containing TGs that are particularly susceptible to peroxidation, in LDs which accumulate in the absence of PRDX6.

## Discussion

In this work, we demonstrate that when PRDX6 is eliminated using CRISPR/Cas9 technology, SNU475 hepatocellular carcinoma cells greatly modify their lipid composition causing a drastic decrease in DHA, an increase in TAGs that accumulate in LDs, a likely disassembly of lipid rafts and a disruption of plasmalogens homeostasis resulting in a higher sensitivity to the induction of ferroptosis. These distinct alterations are the consequence of the loss of the peroxidase, PLA2 and LPCAT activities of PRDX6.

### Sphingomyelins, DHA and lipid rafts

We observed that cells lacking PRDX6 present a selective decrease in all DHA containing glycerophospholipids including DHA itself. PPARα-dependent down-regulation of peroxisomal β-oxidation can result in the very low levels of DHA. PRDX6-dependent PPARα activation has been described [19] and peroxisome proliferator-activated receptor gamma co-activator-1β (PPARGC1B) was predicted with high confidence to be inhibited in SNU475^PRDX6−/−^ cells [16]. The inactivation of PPARα and failure to up-regulate its target gene peroxisomal acyl-CoA oxidase (ACOX1) required for the synthesis of DHA [32,33] would partly explain the observed low levels of DHA.

The specificity of PRDX6 peroxidase activity towards peroxidized DHA should be considered. Transformation of DHA into oxidized species that cannot be detected by our lipidomic protocol could also explain the decrease in DHA since PUFAs are prone to peroxidation under oxidative conditions that prevail in the absence of PRDX6 lipid peroxidase activity [16].

DHA containing membrane glycerophospholipids are incompatible with saturated fatty acids, including cholesterol and provoke their segregation into raft domains increasing their size and stability [34–36]. These domains have been described as signaling hubs in cancer survival/death and invasion [6,34]. It is expected that the drastic decrease in sphingomyelins and DHA provoked by loss of PRDX6 could contribute to destabilization of lipid rafts, although further experimental evidence would be needed to confirm this and its possible relevance in the context of cancer treatment [6].

### Lipid droplets

The role of LD in cancer is not clear, they have been assigned both protumorigenic and antitumorigenic functions [37], although in the case of the hepatocellular carcinoma cells studied here, LD should have an antitumorigenic role since their abundance is coincident with growth arrest. Likewise, the connection between LDs, mitochondria and endoplasmic reticulum (ER) and their involvement in cell death programs through apoptosis-induced mitochondrial dysfunction has also been described [38]. The prominent increase of LD observed in SNU475^PRDX6−/−^ cells should contribute to storage of FA from salvage processes and culture medium [37], but not from *de novo* synthesis since the enzymes of this pathway are lacking in SNU475^PRDX6−/−^ cells [16].

### Preference of PRDX6 for sn2-AA containing PC and role of plasmalogens

The observed changes in glycerophospholipids are coherent with the loss of PLA2 activity of PRDX6 with substrate specificity for PC containing stearic acid at sn1 position and long chain PUFA at sn2. Substrate specificity of PLA2 enzymes are not always clear and there are several isoforms that can distinguish between the various *sn-1* bonds in glycerophospholipids, either acyl or alkyl [39,40].

Plasmalogens, which contain a vinyl ether linkage are also called plasmenyl lipids, are an abundant group of lipids in animals [41,42]. Most plasmalogens possess an ester-linked polyunsaturated fatty acyl (PUFA) chain at the sn-2 position and their biosynthesis involves enzymatic reactions present in both, ER and peroxisomes. An important function of ether-linked membrane phospholipids is AA storage: PLA2-mediated release of AA directly impacts signaling inflammatory processes providing the precursor for many bioactive inflammatory mediators like eicosanoids [41,42]. The eicosanoid derived from AA, 20-hydroxy-eicosatetranoic acid (20-HETE), has been reported to increase invasive and migratory behavior of breast cancer cells, anti-20-HETE therapy is a promising therapeutic approach to reduce tumor size as well as pro-angiogenic patterns in breast tumors [43]. Consequently, a reduction of AA release provoked by the absence of PRDX6 iPLA2 activity could lead to similar anti-20-HETE therapeutic effects.

Eicosanoids share metabolic pathways and regulatory mechanisms with LPC and its derivative lysophosphatidic acid (LPA) [44] affecting various physiological processes, including cell proliferation, survival, apoptosis, cytoskeletal construction, inflammation and cancer [45]. LPA, an indirect product of PRDX6 iPLA2 activity, has been reported to be mediator of the action of PRDX6 in signaling pathways [46] and we have also previously detected inhibition of the regulatory role of LPC in SNU475^PRDX6−/−^ cells [16]. Hence, a dual mechanism derived from the products of the iPLA2 activity of PRDX6, AA and LPC, seems to be the basis of the cellular response to loss of PRDX6.

The correlation between the lack of PRDX6 and the accumulation of AA containing plasmalogens in SNU475^PRDX6−/−^ cells suggest that these lipid molecules are also substrates of PRDX6 PLA2 activity. The increase in plasmalogens content in SNU475^PRDX6−/−^ hepatocarcinoma cells is highly relevant considering that plasmalogens are not abundant in normal liver tissue [47].

### Sensitivity to ferroptosis

SNU475 cells devoid of PRDX6 have increased sensitivity to erastin-induced ferroptosis, but treatment with Fst-1 rescues the cells to the normal erastin-resistant phenotype similar to controls. The peroxidase activity of PRDX6 must be crucial in this antiferroptotic role of PRDX6 since the mechanism of erastin is based on the production of lipoperoxides that are blunted by Fst-1 or reduced by PRDX6. However, inhibition of iPLA2β activity in tumor cells has been reported to induce GPX4-independent ferroptosis [18] and iPLA2 activity of PRDX6 was a negative regulator of ferroptosis [48]. Therefore, not only the lack of the peroxidase activity of PRDX6 might be responsible for the higher sensitivity of SNU475^PRDX6−/−^ cells to undergo ferroptosis, but the absence of iPLA2 activity of PRDX6 might also play a role.

Furthermore, the role of PRDX6 appears critical for the sensitivity of SNU475^PRDX6−/−^ cells to ferroptosis independently of GPX4, as this sensitivity cannot be overcome by increased selenium administration, which also does not affect GPX4 expression levels in the presence or absence of PRDX6. A recent publication demonstrated that PRDX6 acts as a selenium donor for selenophosphate synthetase 2 (SEPHS2) [49] but did not reject the functioning of the ‘classical’ pathway involving selenocysteine lyase (SCLY). Hence, in our cells the PRDX6-dependent pathway for SeCys metabolism and selenoproteins’ synthesis does not seem to be relevant compared with the ‘classical’ pathway involving SCLY. However, our results indicate that in SNU475 hepatocarcinoma cells, PRDX6 plays a more prominent role for elimination of lipid peroxides and ferroptosis prevention compared with GPX4.

The increased levels of transferrin receptor, TFRC in SNU475^PRDX6−/−^ cells that we previously demonstrated [16] could contribute to the enhanced sensitivity to ferroptosis by enhanced iron uptake [50] and plasmalogens could be involved as has been put forward [51–53]. The increase in PUFA containing TAG and accumulation of LD indicates that the cells have initiated a defensive strategy against lipoperoxides by sequestering them into LD [54], although they are still vulnerable and prone to ferroptosis.

Hence, modulation of plasmalogen homeostasis, including their biosynthesis, degradation and turnover, could influence the cell sensitivity to ferroptosis and would partly explain the results observed in SNU475-PRDX6^−/−^ cells. Under normal conditions, besides its peroxidase activity, the putative iPLA2 activity of PRDX6 on plasmalogens would be another way to protect the cell from ferroptosis and would contribute to the release of AA as a precursor for eicosanoids biosynthesis with consequences on signaling pathways.

Ferroptosis inducers (FINs) have been put forward as attractive agents for designing new antitumoral therapies [55]. To target and potentiate the effect of FINs on tumor cells, several strategies including nanoparticles have been developed. Here, we describe PRDX6 knockdown as a potential FIN, reinforcing its nomination as a target to sensitize tumor cells to ferroptosis and to complement and potentiate conventional cancer therapies [55]. Thus, inhibition of PRDX6 expression by nanoparticles containing specific interfering RNA combined with radiotherapy, chemotherapy or immunotherapy could be a hopeful therapy to boost ferroptosis induction and potentiate traditional therapy effectiveness. In this regard, we are conducting promising studies using functionalized nanoliposomes targeting hepatocarcinoma cells containing RNA interference against PRDX6 in combination with chemotherapy.

### Conclusions

The differential lipidomic analysis of SNU475 hepatocarcinoma cells devoid of PRDX6 reveals for the first time drastic changes in lipid composition and cellular distribution providing new clues on the function and specificity of PRDX6 enzymatic activities. DHA deprivation, likely due to its accelerated conversion into oxidized species would indicate a preference for DHA as substrate of PRDX6 lipoperoxidase activity. Accumulation of lipoperoxides leads to GPX4-independent ferroptosis that would be the basis of the reported antitumoral effect of eliminating PRDX6.

**Figure 6.**
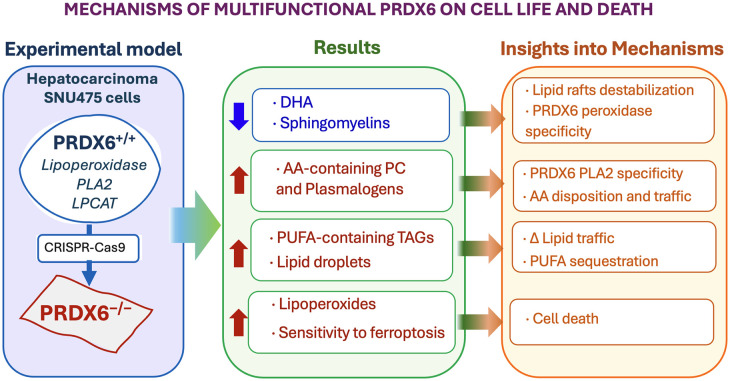
Mechanisms of multifunctional PRDX6 on cell life and death. A visual summary of the article content is shown.

The prominent sphingomyelin decrease could contribute to destabilization of lipid rafts while the loss of ACs correlates with known mitochondrial dysfunction and indicates decreased mitochondrial flux of acyl-CoA, likely responsible for the specific accumulation of PUFA containing TAGs and increase in LDs. Moreover, the exceptional increase in sn2-AA containing phosphatidylcholine and plasmalogens, despite a prominent decrease of all other kinds of glycerophospholipids, suggests specificity of PRDX6 PLA2 activity towards these compounds. This activity would provide PRDX6 with a role in the release of AA as precursor of eicosanoids biosynthesis with effects on eicosanoid signaling pathways (Figure 6).

The results presented highlight the need for further research to establish the role of lipid rafts and LD in cancer and their relationship with the multifunctional role of PRDX6, including the connection of PRDX6 with eicosanoid signaling and the specificity of PRDX6 lipoperoxidase and iPLA2 enzymatic activities.

A visual summary of the article is presented in Figure 6.

## Materials and methods

### Materials and reagents

All reagents were of analytical grade and were purchased from Sigma–Aldrich (St. Louis, MO, U.S.A.) unless otherwise specified. Cell culture dishes and flasks were from TPP (Switzerland).

### Cell lines

SNU475 (CRL-2236) standard cell line was obtained from American Type Culture Collection (LGC Standards, S.L.U., Barcelona, Spain) and it was used as a control (SNU475^PRDX6+/+^ or PRDX6-WT). The SNU475^PRDX6−/−^ (or PRDX6-KO) was generated using CRISPR/Cas9 technology as previously described [16]. They were grown in aseptic conditions and pH 7.4 in an incubator with a 5% CO_2_ atmosphere and 37°C using RPMI 1640 (Gibco) media supplemented with 10% fetal bovine serum and 100 U/l penicillin, 100 μg/ml streptomycin, 0.25 μg/ml amphotericin. Cell free extracts were prepared following detachment with trypsin-EDTA (BioWest).

### Global lipidome analysis

The lipidomic analyses were performed at OWL Metabolomics (Bizkaia, Spain). The samples from six independent experiments were prepared following the company specific guidelines. Briefly, dry cell pellets containing 2 × 10^6^ cells were immediately frozen in liquid nitrogen and stored at −80°C until shipment. Metabolite extraction was accomplished by fractionating the samples into pools of species with similar physicochemical properties, using appropriate combinations of organic solvents. Cell pellets were resuspended in cold water and briefly mixed. Proteins were precipitated from the lysed cell samples by adding a cold solution of chloroform: methanol spiked with metabolites not detected in unspiked cell extracts (internal standards). Samples were incubated for 30 min at −20°C and then vortex mixed and centrifuged at 18 000 × ***g*** for 15 min at 4°C.

Two separate UHPLC-MS platforms optimized for extensive coverage of the lipidome were used [56].

- *Platform 1: Fatty acyls, bile acids, steroids and lysoglycerophospholipids profiling*. 1000 µl of supernatant was collected from samples and dried under vacuum. The dried extracts were reconstituted in methanol before being transferred to vials for UHPLC-MS analysis.
- *Platform 2: Glycerolipids, cholesteryl esters, sphingolipids and glycerophospholipids profiling*. Cold water was added to 200 µl of the supernatants. Then the sample was incubated for 30 min at −20°C. Samples were centrifuged at 18 000  × ***g*** for 15 min at 4°C to facilitate the separation of the organic and aqueous phases. The organic layer was collected, dried under vacuum and resuspended in acetonitrile/isopropanol (1:1) and transferred to vials for UHPLC-MS analysis.

Additionally, different types of quality control (QC) samples were included to assess the data quality [57] and for each analytical platform, randomized sample injections were performed, with each of the QC calibration and validation extracts uniformly interspersed throughout the entire batch run. Chromatography was performed using ACQUITY UPLD systems (Waters Corp., Milford, U.S.A.) for all platforms. A LCT Premier XE Time-of-Flight (ToF) (Waters Corp.) and a Xevo G2-XS QTof (Waters Corp.) mass spectrometers were used for the Lipidomic Platform 1 and 2, respectively. All data were processed using the TargetLynx application manager for MassLynx 4.1 software (Waters Corp.).

Normalization factors were calculated for each metabolite by dividing their intensities in each sample by the recorded intensity of an appropriate internal standard, following the procedure described by Martinez-Arranz et al. [58]. Multivariate data analysis was performed by principal components analysis (PCA) [59] where the data matrix was reduced to a series of principal components (PCs), each a linear combination of the metabolite peak areas. Other statistical analyses performed were Shapiro–Wilk test, Student's *t*-test and Wilcoxon test, and Volcano plot (see Appendix 1 for further details).

### Immunofluorescence

SNU475 cells (1.5 × 10^4^) were seeded on cover glass for fluorescence microscopy with regular medium and allowed to grow overnight. Cells were fixed with 4% paraformaldehyde in PBS solution for 20 min at room temperature and were washed with PBS for 10 min twice. Permeabilization was performed in 0.3% Triton X-100 in PBS solution for 10 min at room temperature. For detection of LD, Nile red solution, 1 µM in PBS, was added and incubated for 90 min at room temperature. Mitochondria were labeled with anti-MTCO2 (Abcam), 1:200 in PBS with 2% BSA overnight at 4°C and incubated for 90 min with secondary antibody (anti-Ig-Mouse Alexa Fluor 488 nm, Abcam), 1:1000 in PBS with 2% BSA. For detection of cytoskeleton the cells were incubated for 90 min at room temperature with a phalloidin dilution (Phalloidin-iFluor 488 Reagent, Abcam), 1:1000 in PBS with 2% BSA. Cells were washed with PBS for 5 min three times and preparations were mounted with mounting medium containing DAPI (Fluoroshield Mounting Medium With DAPI, Abcam). Cells were visualized using a conventional fluorescence microscope Leica DM6B (Wetzlar, Germany) or a spectral confocal microscope ZEISS LSM70. In both cases, analysis and quantification were performed using the FIJI software (ImageJ v1.54f).

### Transmission electron microscopy

Preparation of samples for transmission electron microscopy was carried out at the SCAI of the University of Córdoba. 2 × 10^6^ cells were harvested and fixed with 2.5% v/v solution of glutaraldehyde in 0.1 mol/l phosphate buffer, pH 7.4. Cells were fixed for 30 min in a 1% osmium tetroxide solution in the same buffer, dehydrated using increasing concentrations of ethanol, and embedded in Epon resin. 90 nm sections were cut using an ultramicrotome, stained with 5% uranyl acetate and 5% lead citrate, and analyzed on a JEM1400 transmission electron microscope at 80 kV.

### Plasmid transfection

The pcDNA3.1 plasmid containing PRDX6 cDNA or GFP cDNA was used to transfect SNU475^PRDX6−/−^ or SNU475^PRDX6+/+^ cells respectively. 2 × 10^5^ cells were seeded in a 6 wells plate and were transfected for 6 h upon reaching 80% of confluence. Transfection was performed using the lipofectamine 3000 kit according to manufacturer guidelines, using 1.2 µg or 2.4 µg of plasmid DNA and a ratio of 1:2:2 [DNA (µg): P3000 reagent (µl): lipofectamine (µl)]. Transfection efficiency was measured in cells transfected with GFP cDNA using fluorescence microscopy and by Western blot.

### Cell lysis, protein quantification, SDS–PAGE and Western blot

Cells were lysated at 4°C with RIPA lysis buffer containing protease and phosphatase inhibitors (SIGMA) and supernatant collected. Protein concentration was determined using a BCA Assay Kit (Thermo Fisher Scientific) with BSA as the standard. SDS–PAGE was performed in 12% or 15% acrylamide gels and electrophoresis was carried out in a Mini-protean Tetra Cell system (Bio-Rad) for 90 min at 120 V. After electrophoresis, proteins were transferred to a nitrocellulose membrane using a wet transfer system for 75 min at 300 mA. Transfer and protein load were checked by staining with Ponceau reagent. Membranes were blocked with 5% non-fat milk in TBS-T for 2 h at room temperature and then incubated overnight at 4°C with the primary antibodies anti-PRDX6 (ProteinTech) at 1:4000 dilution or anti-GPX4 (Sigma–Aldrich) at 1:1000 dilution in 1% non-fat milk in TBS-T. Membranes were washed with TBS-T and incubated with peroxidase-conjugated anti-rabbit secondary antibody used at 1:4000 dilution for at 1 h at room temperature. The chemiluminescent signal produced by the ECL reagent (Bio-Rad) was detected on a ChemiDoc image analyzer (Bio-Rad) and quantified with ImageJ software using Ponceau's staining as a reference for protein normalization.

### Measurement of lipid peroxidation

Lipid peroxidation was quantified by fluorescence (*λ*_ex_ = 525 nm; *λ*_em_ = 550 nm) using TBARS Assay Kit (Canvax Biotech S.L., Cordoba, Spain), based on the reaction of malondialdehyde (MDA) derived from peroxidized lipids with thiobarbituric acid (TBA). Results were normalized by cell counting.

### Cell survival assay

Total number of cells and cell viability in a SNU475 cell suspension were quantified using the trypan blue dye exclusion method*.* Cell survival was also determined using the SRB assay as described in [12] following treatments with erastin (0, 2, 3 and 4 µM) and ferrostatin-1 (0, 10 µM), both dissolved in DMSO. Prior to treatments, cells were cultured or not with sodium selenite (100 nM) for 48 h. Cells grown to 80–90% confluence were fixed with 3.3% (w/v) trichloroacetic acid at 4°C for 1 h, washed several times with water and stained with 0.057% (w/v) SRB solution in 1% (v/v) acetic acid for 30 min. Excess dye was then removed by washing with 1% (v/v) acetic acid and 200 µl of 10 mM Tris base solution (pH 10.5) was added to each well. Protein-bound dye was determined by measuring absorbance at 510 nm.

### Statistics

Where appropriate, results are expressed as mean ± SD of at least three independent experiments. Statistical analysis of the data was performed by unpaired Student's *t*-test or one/two-way ANOVA, depending on the experiment design, as detailed in the figure legends, with the threshold for statistically significant differences set at *P* adjust <0.05 value. A detailed description of the statistical tests used for the lipidomic approach is included in Appendix 1.

## Data Availability

All data are contained within the manuscript.

## Abbreviations

20-HETE: 20-hydroxy-eicosatetranoic acid
AA: arachidonic acid
AC: acyl-carnitine
Cer: ceramide
Cho: cholesterol
ChoE: cholesteryl ester
CMH: monohexosylceramide
DHA: docosahexanoic acid
ER: endoplasmic reticulum
FA: fatty acid
FIN: ferroptosis inducer
GPX4: gluthathione peroxidase 4
HFA: hydroxy fatty acid
HpFA: fatty acid hydroperoxide
iPLA_2_: calcium-independent phospolipase A_2_
LD: lipid droplet
LPA: lysophosphatidic acid
LPC: LysoPC
LPCAT: lysophosphatidylcholine acyl transferase
LPE: lysoPE
LPI: LysoPI
LPL: lysophospholipids
MDA: malondialdehyde
MUFA: monounsaturated-FA
PC: phosphatidylcholine
PE: phosphatidylethanolamine
PI: phosphatidylinositol
PL: phospholipid
PUFA: polyunsaturated-FA
SFA: saturated-FA
SM: sphingomyelin
TBA: thiobarbituric acid
TFRC: transferrin receptor
TG: triglyceride

## Appendix 1

### Data pre-processing

All data were processed using the TargetLynx application manager for MassLynx 4.1 software (Waters Corp., Milford, U.S.A.). A set of predefined retention time, mass-to-charge ratio pairs, *Rt*-*m/z*, corresponding to metabolites included in the analysis are fed into the program. Associated extracted ion chromatograms (mass tolerance window = 0.05 Da) are then peak-detected and noise-reduced in both the LC and MS domains such that only true metabolite related features are processed by the software. A list of chromatographic peak areas was generated for each sample injection. For identified metabolites, representative MS detection response curves were generated using an internal standard for each chemical class included in the analysis. By assuming similar detector response levels for all metabolites belonging to a given chemical class, allowed a linear detection range to be defined for each variable. Maximum values were defined as those at which the detector response became non-linear with respect to the concentration of the representative internal standard. Variables were not considered for further analysis where more than 30% of data points were found outside their corresponding linear detection range.

### Data analysis

Data pre-processing generated a list of chromatographic peak areas for the metabolites detected in each sample injection. An approximated linear detection range was defined for each identified metabolite, assuming similar detector response levels for all metabolites belonging to a given chemical class represented by a single standard compound. Those metabolites for which more than 30% of data points were found outside their corresponding linear detection range were highlighted, as the interpretation of results based on their data must be carefully considered. Data normalization was performed following the procedure described by Martinez-Arranz et al. [58]. Once normalized, the dimensionality of the complex data set was reduced to enable visualization of any metabolic clustering of the different groups of samples. This was achieved by multivariate data analysis, including the non-supervised principal components analysis (PCA). Multivariate data analyses were performed using the SIMCA software (version SIMCA 14.1. MSK Umetrics AB, Umea, Sweden). Data were centered and unit variance (UV)- scaled. Model quality was assessed using *R*^2^ and *Q*^2^ values, which indicate the explained fraction of variance and the accuracy of prediction, respectively. The *Q*^2^ parameter was calculated by sevenfold cross validation. Univariate statistical analyses were also performed calculating group fold-changes and unpaired Student's *t*-test *P*-value (or Welch's *t*-test where unequal variances were found) for the comparison PRDX6 KO vs. PRDX6 WT samples.

### Distribution of the data

As normal distribution of the data should not be assumed a priori, distribution of each metabolite and chemical class was studied per group. Univariate normality was assessed by the Shapiro–Wilk test. The results of the Shapiro test can be found in the sheet ‘Data per metabolite’ of the Excel file (Supplementary Table S1) or ‘Data per chemical class’ in the case of the calculated chemical classes. Thus, results included in this report were based on parametric results: fold-changes (fold-change of the mean) and Student's *t*-test for the unpaired comparison. However, non-parametric analysis [robust fold-change (fold-change of the medians) and Wilcoxon–Mann–Whitney test (also called the Mann–Whitney *U*-test)] can also be found in the Excel file.

### Multivariate data analysis

The first objective in the data analysis process was to reduce the dimensionality of the complex data set to enable easy visualization of any metabolic clustering of the different groups of samples. This was achieved by principal components analysis (PCA) where the data matrix is reduced to a series of principal components (PCs), each a linear combination of the metabolite peak areas. Each successive PC explains the maximum amount of variance possible, not accounted for by the previous PCs. One type of plot is included in this article: Scores plot displays the samples as situated on the projection planes described by the PCs.

The number of components or model dimensions (A) included in the model is linked to the difference between the degree of fit and the predictive ability (*R*^2^ and *Q*^2^ parameter respectively). *R*^2^ provides an indication of how much of the variation within the data set can be explained by the model (goodness of fit). *Q*^2^ parameter describes the predictive ability of model (goodness of prediction).

The *R*^2^ and *Q*^2^ parameters display entirely different behavior as the model complexity increases (A increases). The goodness of fit, *R*^2^, varies between 0 and 1, where 1 means a perfectly fitting model and 0 no fit at all. However, *R*^2^ is inflationary and approaches to unity as A increases. Hence, it is not sufficient to have a high *R*^2^. On the other hand, the goodness of prediction, *Q*^2^, is less inflationary and will not automatically come close to 1 with increasing A. Additionally, depending on the type of samples included in the analysis the cut-off values might change, but usually if *Q*^2^ value is over the 20% of *R*^2^ it can be consider as a good PCA model.

### Other statistical analyses

#### Shapiro–Wilk test

The Shapiro-Wilk test was used for testing the normality of data. It was applied to each metabolite to detect whether a sample comes from normally distributed population.

#### Student's *t*-test and Wilcoxon test

After normality test, metabolites were evaluated by calculating group percentage changes and Student's *t*-test *P*-value (normal distribution) or Wilcoxon–Mann–Whitney test (also called the Mann–Whitney *U*-test) (non-normal distribution) for the unpaired comparisons included in the study. Similarly, pairwise fold-changes and paired Student's *t*-test *P*-value (normal distribution) or Wilcoxon signed-rank test (non-normal distribution) for the paired comparisons.

#### Volcano plot

The ‘volcano plot’ is an effective and easy-to-interpret graph that summarizes both fold-change and *t*-test or Wilcoxon test criteria. It is a scatter-plot of the negative log_10_-transformed *P*-values test against the log_2_ fold change. Metabolites with statistically significant differential levels according to the *t*-test (or Wilcoxon test) will lie above a horizontal threshold line. Metabolites with large fold-change values will lie far from the vertical threshold line at log_2_ fold change = 0, indicating also if the metabolite is up or down-regulated.

#### Heatmap

The heatmap allows an easy visualization of the significant increments or decreases on the metabolic profile of a group of samples compared with another one. Blue sections of the heatmap denote reduced metabolites (negative log_2_ fold-changes) and red sections denote metabolites increased (positive log_2_ fold-changes) in group B compared with A. Gray/black bars indicate significant *P*-values of the Student's *t*-test or Wilcoxon test (light gray, *P* < 0,05; dark gray, *P* < 0,01; black, *P* < 0,001).
