# Supplementary material for: Loss of peroxiredoxin 6 alters lipid composition and distribution resulting in increased sensitivity to ferroptosis
Source: Biochem J. 2024 Dec 23;481(24):1997–2015. doi: 10.1042/BCJ20240445 (PMC11668489; doi:10.1042/BCJ20240445)
Supplement: Supplementary Figures [file BCJ-481-1997-s1.pdf]

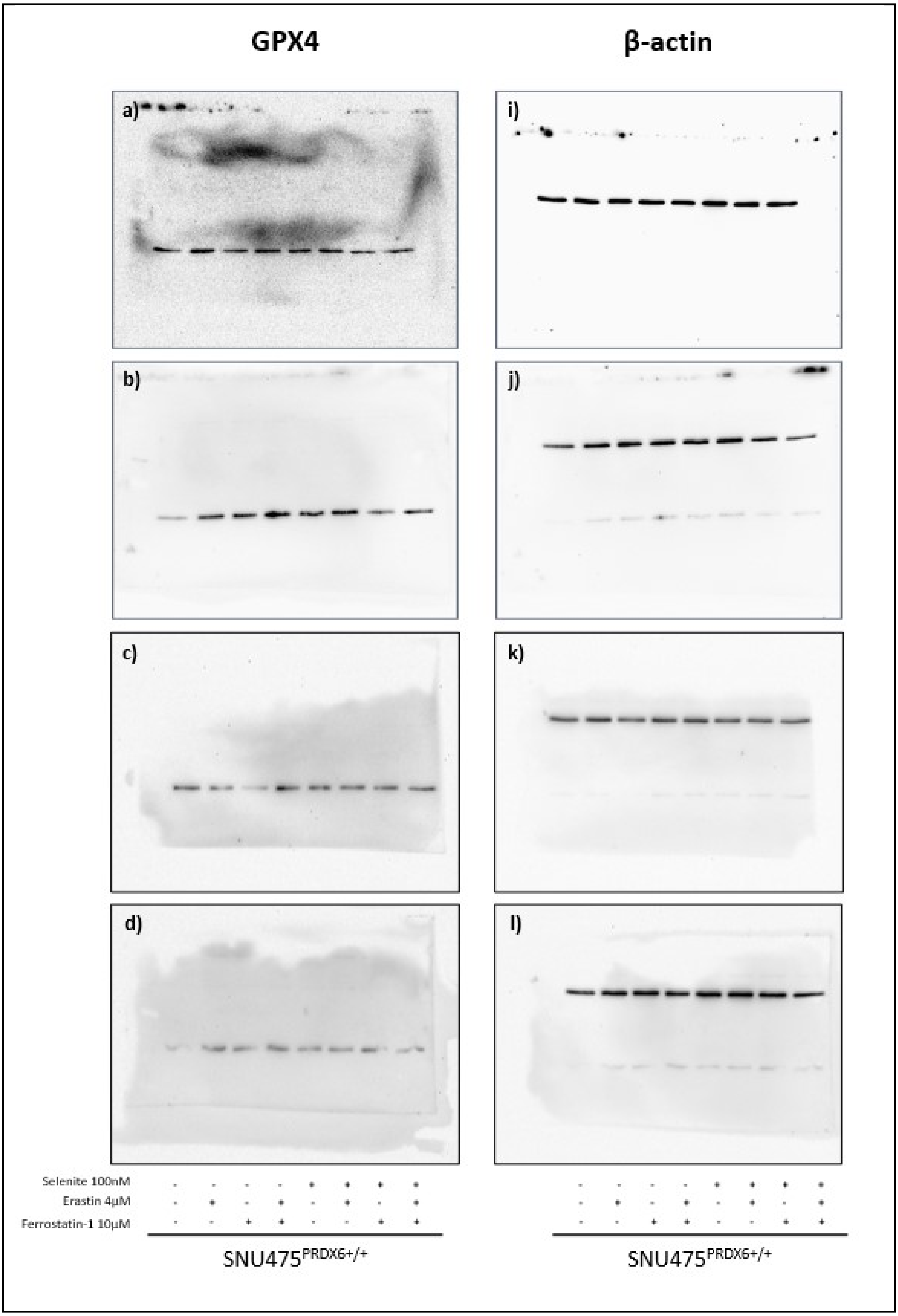
GPX4
β-actin
a)
b)
c)
d)
i)
j)
k)
l)
Selenite 100nM
Erastin 4μM
Ferrostatin-1 10μM
SNU475PRDX6+/+
SNU475PRDX6+/+

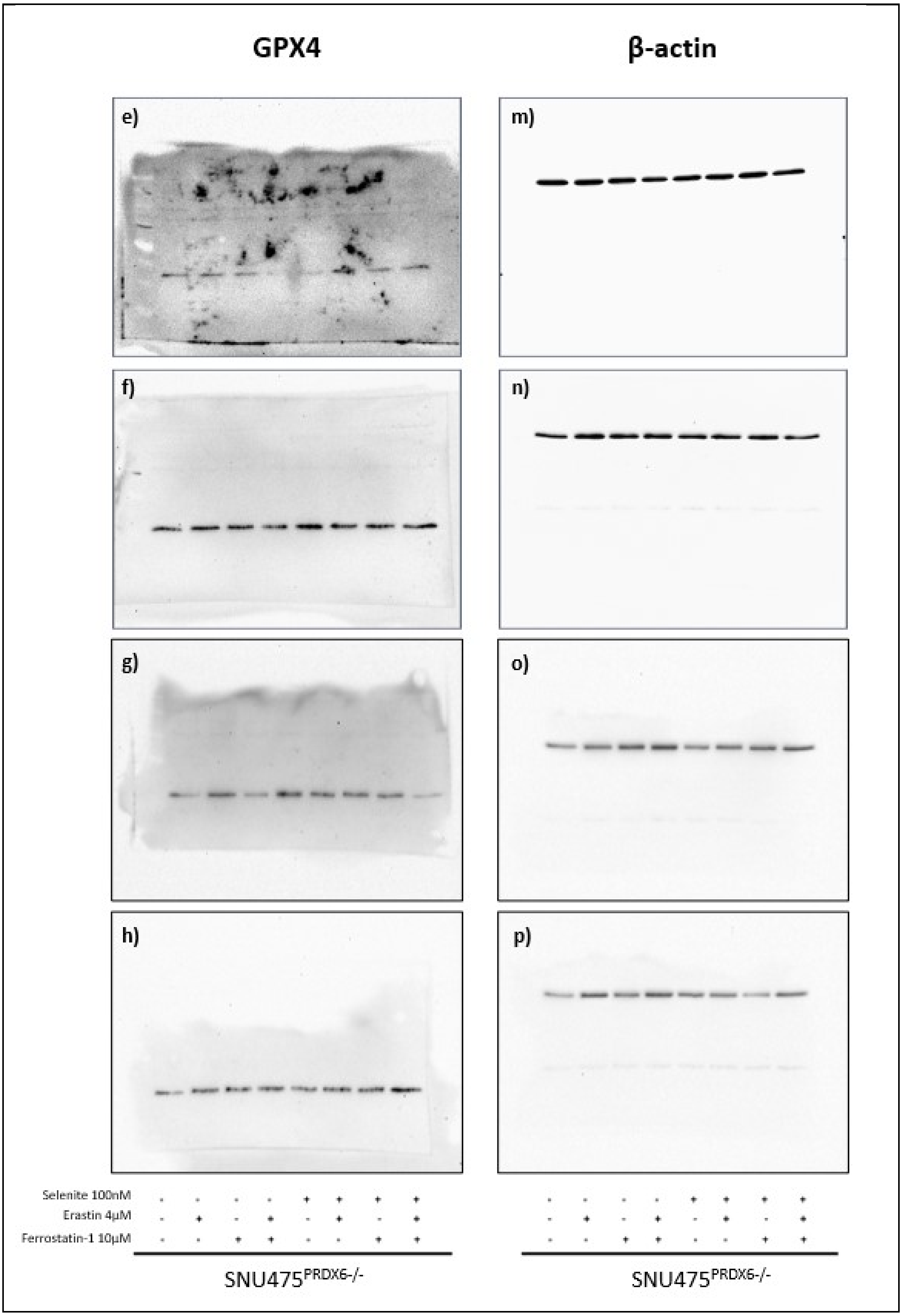
GPX4
β-actin
e)
f)
g)
h)
m)
n)
o)
p)
Selenite 100nM - - - - + + + +
Erastin 4μM - + - + - + - +
Ferrostatin-1 10μM - - + + - - + +
SNU475PRDX6-/-
SNU475PRDX6-/-

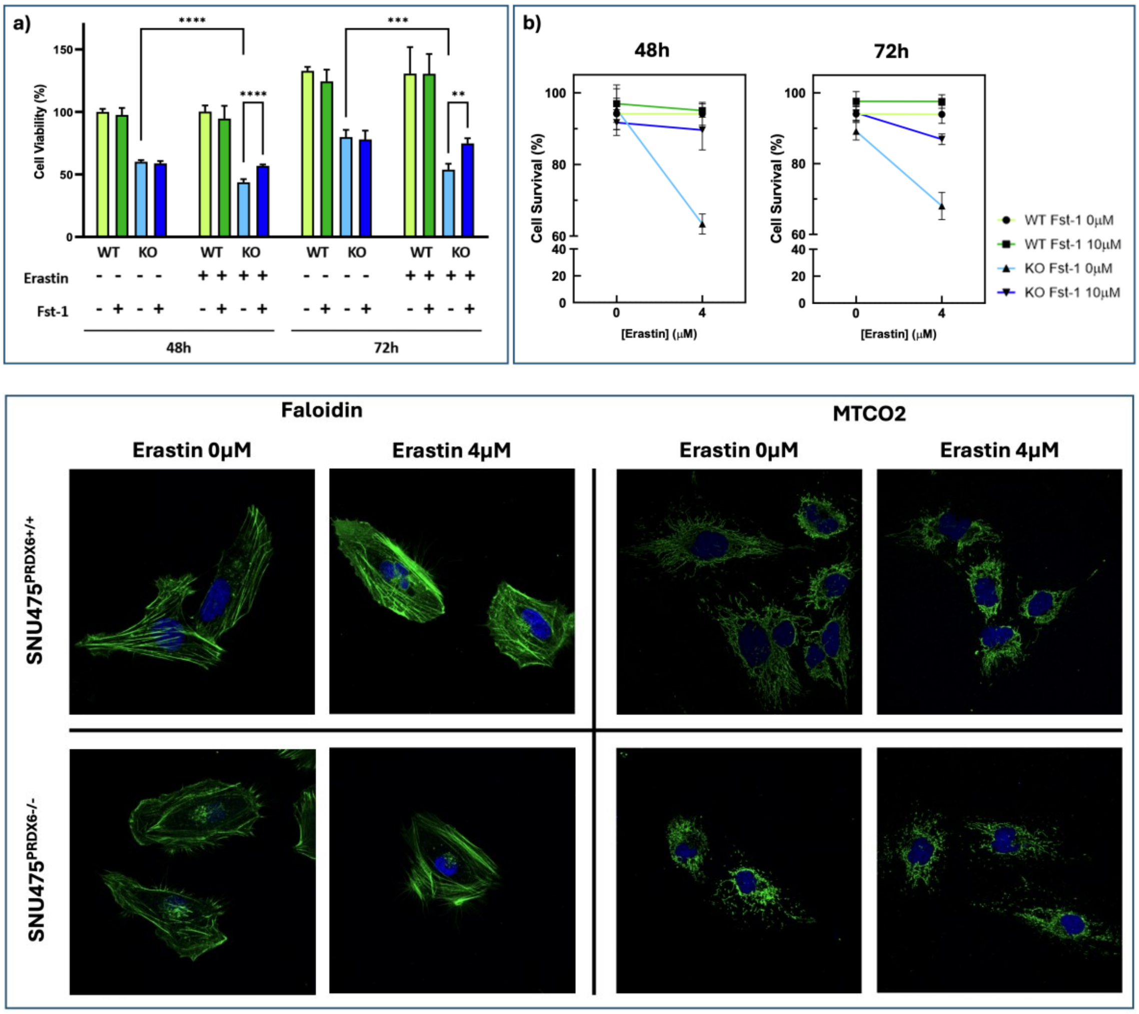
a)
Cell Viability (%)
150
100
50
0
****
****
***
**
WT KO WT KO WT KO WT KO
Erastin
Fst-1
48h
72h
b)
48h
72h
Cell Survival (%)
100
80
60
40
20
0
[Erastin] (μM)
WT Fst-1 0μM
WT Fst-1 10μM
KO Fst-1 0μM
KO Fst-1 10μM
Faloidin
MTCO2
Erastin 0μM
Erastin 4μM
Erastin 0μM
Erastin 4μM
SNU475PRDX6+/+
SNU475PRDX6-/-
